# A Study of 29 Pediatric Patients With Osteomyelitis Resulting From Neuropathy and Pressure Effects

**DOI:** 10.1155/jotm/9967043

**Published:** 2026-09-23

**Authors:** Fatma Kılınç, Akif Mirioğlu, Derya Alabaz, Ümmühan Çay, Özlem Özgür Gündeşlioğlu

**Affiliations:** ^1^ Pediatric Infectious Diseases Department, Niğde Ömer Halisdemir University Training and Research Hospital, Niğde, Türkiye; ^2^ Department of Orthopedics and Traumatology, Çukurova University, Faculty of Medicine, Adana, Türkiye, cu.edu.tr; ^3^ Pediatric Infectious Diseases Department, Çukurova University, Faculty of Medicine, Adana, Türkiye, cu.edu.tr

**Keywords:** children, neuropathy, osteomyelitis

## Abstract

**Background:**

Pediatric osteomyelitis secondary to underlying neuropathies is a rare, complex, but clinically severe entity. Loss of protective pain sensation and proprioception predisposes children to repetitive unperceived microtrauma and chronic pressure ulceration, facilitating insidious progression to deep bone infection. Comprehensive pediatric studies documenting the presentation, diagnostic characteristics, microbiological spectrum, and long‐term outcomes of this vulnerable cohort remain exceptionally scarce.

**Objective:**

To characterize the clinical presentation, inflammatory biomarker utility, microbiological profile, surgical intervention rates, and disease recurrence patterns in pediatric patients with neuropathy‐associated osteomyelitis managed at a tertiary referral center.

**Methods:**

This single‐center descriptive case series analyzed 29 pediatric patients (< 18 years) with confirmed underlying neuropathies and MRI/pathologically proven chronic osteomyelitis between 10.10.2012 and 20.09.2022. Clinical features, laboratory markers, microbiological isolates, operative procedures, hospitalizations, and long‐term recurrence rates were extracted and evaluated.

**Results:**

The cohort comprised 15 males (51.7%) and 14 females (48.3%) with a median age at presentation of 122 months (range: 31–211 months). Underlying neuropathies included myelomeningocele (69.0%), hereditary sensory and autonomic neuropathies (27.6%), and cerebral palsy (3.4%). Overlying cutaneous ulcers were present in 89.7% of cases, with a marked mean diagnostic delay of 22.8 ± 17.8 months (median: 24 months). ESR (65.5%) and CRP (75.9%) demonstrated high diagnostic sensitivity, whereas procalcitonin was elevated in only 10.3%. Microbiological analysis revealed a complex polymicrobial profile yielding 42 distinct isolates across 89.7% of cultured patients. Surgical intervention was required in 86.2% of patients, with 37.9% undergoing lower extremity amputation. The median hospital stay was 36.0 days (range: 1–333 days). Among patients completing regular outpatient follow‐up, 100% experienced chronic relapses requiring multiple re‐admissions (up to 14 admissions).

**Conclusions:**

Factors such as resistant microorganisms, multiple antibiotic use, prolonged hospitalizations, the financial burden on the healthcare sector, and deterioration in patients’ quality of life show the importance of preventive measures for this disease group that progresses with chronic and recurrent osteomyelitis.

## 1. Introduction

Osteomyelitis, one of the most common musculoskeletal system infections in childhood, is a severe health problem even in developed countries [1]. Incidence is reported as 1.2–13/100,000 per year in the pediatric population [2]. However, osteomyelitis cases have been indicated to increase in recent years, and their incidence varies in different countries. The highest rate of osteomyelitis reported worldwide was 82/per 100,000 in Indigenous children in Northern Australia, compared to 9/100,000 in non‐Indigenous children. The incidence rate of osteomyelitis (nonvertebral) is calculated as 10/100,000 children in Norway and 7.8–9.1/100,000 children in South Korea. Hospitalization rates for acute osteomyelitis in the United States range from 1.34 to 1.66 per 100,000 children. A study conducted in Germany observed that the incidence rates of osteomyelitis in children increased by 11.7% from 8.2/100,000 in 2009 to 9.2/100,000 in 2019 [1]. There are independent studies in Turkey, but there are no precise data.

Bone tissue is accepted as relatively resistant to the settlement of microorganisms. Situations that cause direct invasion due to bacteremia or trauma in childhood facilitate the formation of osteomyelitis [3]. In addition, it is known that the feeling of pain is an important mechanism that protects the body and tissues against trauma, and the neuropathy that may develop (lack of pain sensation) paves the way for trauma. [4]. Our study significantly contributes to the nascent literature on osteomyelitis in neuropathic pediatric patients. While osteomyelitis in children is a well‐recognized health problem with reported incidences, studies specifically detailing the characteristics, course, and management outcomes in children with underlying neuropathies are notably scarce. Our cohort of 29 patients, predominantly with myelomeningocele and hereditary neuropathies, offers valuable insights into this vulnerable population, bridging a critical gap in current understanding. We aim to share the results of osteomyelitis cases that develop because of physical trauma in patients with neuropathy and to raise awareness that this result can be reduced by taking precautions.

## 2. Materials and Methods

### 2.1. Study Design and Setting

This was a retrospective, single‐center, descriptive case series conducted at Çukurova University Hospital. The study period was from 10.10.2012 to 20.09.2022, allowing a minimum potential follow‐up window of at least 12 months for patients diagnosed at the end of the inclusion window. The unit of analysis was the patient; for patients with multiple osteomyelitis episodes, the first episode served as the index event. The study is reported in accordance with the Strengthening the Reporting of Observational Studies in Epidemiology checklist for descriptive observational studies.

### 2.2. Study Population

Inclusion criteria: Patients were eligible if they met all of the following at the time of the index osteomyelitis episode:

⒈ Age < 18 years at the time of diagnosis.
⒉ A confirmed underlying neuropathic condition—operated myelomeningocele/spina bifida, hereditary sensory and autonomic neuropathy including congenital insensitivity to pain with anhidrosis, or another neuropathy documented in the medical record.
⒊ Osteomyelitis confirmed by (i) magnetic resonance imaging (MRI) findings consistent with osteomyelitis and/or histopathological demonstration of chronic osteomyelitis and (ii) a clinical course compatible with bone infection.

Exclusion criteria. (i) patients with incomplete documentation of either the neuropathic diagnosis, the imaging/pathology confirmation of osteomyelitis, or basic demographic information; (ii) patients with acute hematogenous osteomyelitis without underlying neuropathy or pressure injury; and (iii) patients with isolated soft tissue infections without bone involvement were excluded.

### 2.3. Diagnostic Definitions

Neuropathy identification: Underlying neuropathic conditions were identified based on standardized clinical examinations, neuroimaging, electroneuronography, and/or genetic testing documented in patient records by pediatric neurologists or geneticists prior to or during admission.

Osteomyelitis definition: Acute osteomyelitis was defined as symptomatic bone infection of short duration (< 2 weeks) without radiological evidence of bone necrosis or sequestrum formation. Chronic osteomyelitis was operationalized as long‐standing infection (symptoms typically > 2–4 weeks or presenting with chronic ulceration/sinus tract) characterized by necrotic bone (sequestrum), involucrum formation on MRI, and/or histopathological confirmation of chronic inflammatory changes from intraoperative tissue biopsies.

Polymicrobial infection was defined as the isolation of ≥ 2 distinct microbial species from the same episode.

### 2.4. Statistical Analysis

Statistical analyses were performed using IBM SPSS Statistics for Windows, Version 26.0 (IBM Corp., Armonk, NY, USA). Given the descriptive nature of this single‐group case series, no inferential statistical testing (no hypothesis‐driven *p* values) was performed. Continuous variables are reported as mean ± standard deviation with 95% confidence intervals where applicable; medians and interquartile ranges are reported when distributions show appreciable skewness, particularly for the interval between lesion onset and admission, length of hospital stay, and number of hospitalizations. Categorical variables are reported as counts and percentages with Wilson 95% confidence intervals to provide clinically meaningful precision around small proportions, as recommended for descriptive case‐series reporting.

### 2.5. Ethics

The study protocol was reviewed and approved by the Çukurova University Hospital Ethics Committee (Approval number: 145/14.06.2024). Because this was a retrospective analysis of records from patient care already provided, the requirement for individual parental informed consent was waived by the committee, in accordance with national regulations governing retrospective chart‐review studies. All patient data were anonymized at the point of extraction, stored in a password‐protected file accessible only to investigators, and handled with strict confidentiality throughout the analysis. No identifying images are published. This study did not receive any specific grant from funding agencies in the public, commercial, or not‐for‐profit sectors.

## 3. Results

The retrospective study included 29 patients: 15 males (51.7%, Wilson 95% CI: [34.4%–68.6%]) and 14 females (48.3%, Wilson 95% CI: [31.4%–65.6%]). The mean age at presentation was 120.3 ± 51.9 months (95% CI: 100.6–140.1 months), with a median age of 122 months (IQR: 77–154 months; range: 31–211 months). The median follow‐up was 2 years (IQR: 1–3 years; mean: 2.7 ± 2.5 years, 95% CI: 1.7–3.6 years), with the longest follow‐up extending to 9 years.

Regarding the primary diagnoses, 20 patients (69.0%, Wilson 95% CI: [51.3%–83.1%]) had operated spina bifida (myelomeningocele), 8 (27.6%, Wilson 95% CI: [14.7%–45.2%]) had hereditary neuropathy, and 1 (3.4%, Wilson 95% CI: [0.6%–17.2%]) had cerebral palsy (Table 1). All patients were diagnosed with chronic osteomyelitis based on MRI findings and tissue culture results.

**TABLE 1:** Primary diagnosis of the patient and affected anatomical location.

	No. of patients (%)
Diagnosis	
Spinal dysmorphism	20 (69)
Hereditary neuropathy	8 (27.6)
Cerebral palsy	1 (3.4)
Anatomical location	
Foot and ankle	23 (79.4)
Femur	3 (10.4)
Sacrum	1 (3.4)
Tibia and femur	1 (3.4)
Ankle with tibia or fibula	1 (3.4)

**FIGURE 1:**
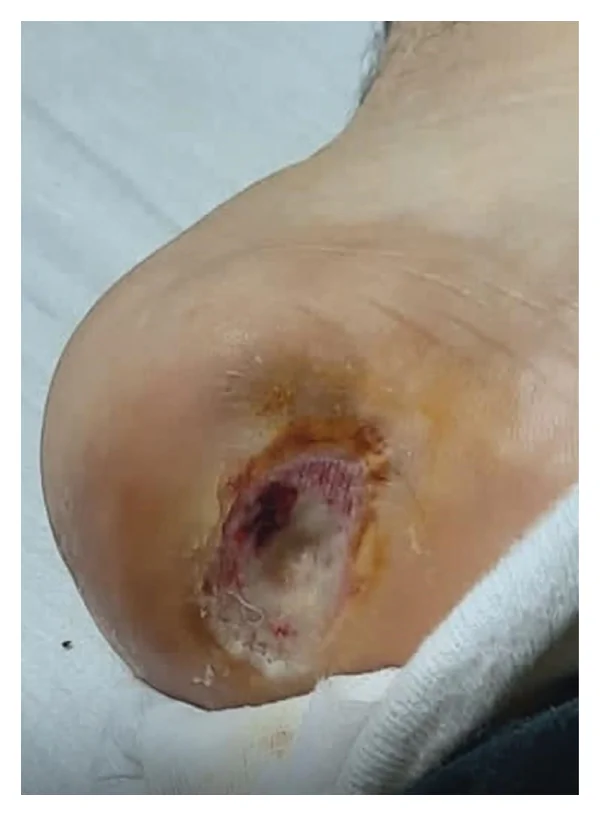
The patient’s ulcerated lesion on the heel at the time of arrival.

Concerning presenting clinical features, local inflammatory signs (hyperemia and edema) were present in all patients; ulcerated lesions were present in 26 patients (89.7%, Wilson 95% CI: [73.6%–96.4%]) (Figure 1), and fever was observed in 12 patients (41.4%, Wilson 95% CI: [25.5%–59.3%]). The mean time between the onset of the soft tissue lesion and hospital admission was 22.8 ± 17.8 months (95% CI: 16.0–29.6 months), with a median duration of 24 months (IQR: 7–36 months; range: 1–60 months).

While only 1 patient (3.4%, Wilson 95% CI: [0.6%–17.2%]) had sacrum involvement, all other patients had lower extremity localizations (Table 1). Laboratory findings at initial admission revealed an elevated erythrocyte sedimentation rate (ESR) in 19 patients (65.5%, Wilson 95% CI: [47.3%–80.1%]; [median: 36.0 mm/h, IQR: 15.0–59.0 mm/h; mean: 44.5 ± 35.2 mm/h]), elevated C‐reactive protein (CRP) in 22 patients (75.9%, Wilson 95% CI: [57.9%–87.8%]; [median: 22.0 mg/L, IQR: 8.3–81.0 mg/L; mean: 48.2 ± 59.3 mg/L]), and elevated procalcitonin in 3 patients (10.3%, Wilson 95% CI: [3.6%–26.4%]; [median: 0.06 μg/L, IQR: 0.03–0.36 μg/L; mean: 3.41 ± 11.5 μg/L]) (Table 2).

**TABLE 2:** Laboratory parameters of the patients.

Laboratory parameter	Minimum	Maximum	Mean
White blood cell (/mm^3^)	4390	18,400	9437.58
Absolute neutrophil count (/mm^3^)	1530	14,700	5354.82
Absolute lymphocyte count (/mm^3^)	1000	5510	3208.96
Hemoglobin (g/dL)	4.5	14.1	10.38
Platelet count (/mL)	186,000	953,000	428,551.72
Vitamin D	3.6	51	18.37
Vitamin B12	113	426	212.75

MRI was performed in 28 patients (96.6%, Wilson 95% CI: [82.8%–99.4%]). Positive blood cultures were detected in 4 patients (13.8%, Wilson 95% CI: [5.5%–30.6%]). Culture samples from ulcerative lesions, abscesses, or deep tissue yielded positive results in 26 patients (89.7%, Wilson 95% CI: [73.6%–96.4%]), with a total of 42 distinct microorganisms isolated (Table 3). No growth was observed in 3 patients (10.3%, Wilson 95% CI: [3.6%–26.4%]). Among these three cases, culture samples were obtained but yielded no growth in two patients, whereas no culture could be obtained in one patient due to the absence of a suitable specimen. Due to this microbial diversity, 24 different systemic antibiotics were utilized during treatment. The most frequently administered parenteral antibiotics were teicoplanin, meropenem, clindamycin, amikacin, and ciprofloxacin. Chronic osteomyelitis treatment was initiated in all patients upon admission. Parenteral therapy was started targeted to the isolated microorganisms, followed by sequential oral or outpatient parenteral antimicrobial therapy.The mean number of hospitalizations per patient was 3.1 ± 3.4 times (95% CI: 1.8–4.4 times), with a median of 2.0 hospitalizations (IQR: 1.0–3.0 times; range: 1–14 times). The overall mean duration of hospital stay was 57.9 ± 66.9 days (95% CI: 32.5–83.4 days), with a median duration of 36.0 days (IQR: 25.0–58.0 days; range: 1–333 days).

**TABLE 3:** Microorganisms isolated through tissue, abscess, and swab cultures.

	Microorganism	*N*	Microorganisms subtype	*N* (%)
Gram‐positive microorganism (*N*: 64)	*Staphylococcus* spp.	37	Methicillin‐resistant	
			*Staphylococcus aureus*	10 (34.5)
			*Staphylococcus haemolyticus*	10 (34.5)
			Methicillin‐sensitive	
			*Staphylococcus aureus*	8 (27.6)
			*Koagülaznegatifstafilakok*	3 (10.3)
			*Coagulase-negative staphylococci*	3 (10.3)
			*Staphylococcus hominis*	2 (6.9)
			*Staphylococcus lugdunensis*	1 (3.4)
	*Streptococcus* spp.	18	*Streptococcus dysgalactia*	10 (34.5)
			*Streptococcus viridans*	3 (10.3)
			*Streptococcus suis*	1 (3.4)
			*Streptococcus pyogenes*	1 (3.4)
			*Streptococcus anginosus*	1 (3.4)
			*Streptococcus constellatus*	1 (3.4)
			*Streptococcus pseudoporcinus*	1 (3.4)
	*Enterococcus* spp.	6	*Enterococcus faecalis*	5 (17.2)
			*Enterococcus faecium*	1 (3.4)
	*Haemophilus* spp.	3	*Haemophilus influenza*	2 (6.9)
			*Haemophilus saphrophilus*	1 (3.4)

*Gram-negative microorganism (N: 67)*	*Pseudomonas* spp.	13	*Pseudomonas aeruginosa*	12 (41.4)
			*Pseudomonas fluorescens*	1 (3.4)
	*Escherichia coli*	10		10 (34.5)
	*Proteus* spp.	8	*Proteus mirabilis*	5 (17.2)
			*Proteus species*	2 (6.9)
			*Proteus vulgaris*	1 (3.4)
	*Morganella morganii*	7		7 (24.1)
	*Acinetobacter* spp.	7	*Acinetobacter baumannii*	6 (20.7)
			*Acinetobacter haemolyticus*	1 (3.4)
	*Klebsiella* spp.	6	*Klebsiella pneumonia*	4 (13.8)
			*Klebsiella species*	1 (3.4)
			*Klebsiella oxytoca*	1 (3.4)
	*Enterobacter* spp.	5	*Enterobacter cloacae*	4 (13.8)
			*Enterobacterhormaechei*	1 (3.4)
	*Achromobacter* spp.	4	*Achromobacter xylosoxidans*	2 (6.9)
			*Achromobacter denitrificans*	2 (6.9)
	*Pasteurella* spp.	2	*Pasteurella canis*	1 (3.4)
			*Pasteurella pneumotropica*	1 (3.4)
	*Eikenella corrodens*	2		2 (6.9)
	*Providencia rettgeri*	1		1 (3.4)
	*Oligella ureolytica*	1		1 (3.4)
	*Stenetrophomonas maltophilia*	1		1 (3.4)

Fungi:3	*Candida* spp.	3	*Candida albicans*	2 (6.9)
			*Candida nonalbicans*	1 (3.4)

Surgical treatment was performed in 25 patients (86.2%, Wilson 95% CI: [69.4%–94.5%]), and 11 patients (37.9%, Wilson 95% CI: [22.7%–55.9%]) underwent amputation (Table 1). All pathological specimens demonstrated chronic nonspecific inflammation. Over the long‐term follow‐up period, 18 patients (62.1%, Wilson 95% CI: [44.1%–77.3%]) were lost to follow‐up. Among the 11 patients (37.9%, Wilson 95% CI: [22.7%–55.9%]) who continued regular outpatient follow‐up, all experienced recurrent hospitalizations due to chronic osteomyelitis relapse. Specifically, two patients were hospitalized 14 times, one 7 times, four 4 times, one 3 times, and three 2 times. Outpatient follow‐up for these active cases remains ongoing.

## 4. Discussion

This study describes the presentation, diagnostic findings, microbiological profile, and management outcomes of 29 pediatric patients with osteomyelitis secondary to underlying neuropathies, predominantly operated spina bifida (69.0%) and hereditary neuropathies (27.6%). Our findings highlight a distinct clinical presentation defined by a high prevalence of overlying ulcerative lesions (89.7%) and a profound delay in presentation, with a mean interval of 22.8 months (median: 24 months) between lesion onset and hospital admission. This delay is substantially longer than the average 10‐month evaluation delay reported in general pediatric chronic osteomyelitis cohorts [5]. In children with loss of protective pain sensation and proprioception, repetitive unperceived trauma allows superficial pressure ulcers to insidiously progress to deep bone infection, primarily affecting the load‐bearing foot and ankle region (79.3%) [4, 6–9].

Regarding inflammatory biomarkers, elevated ESR (65.5%) and CRP (75.9%) served as reliable diagnostic indicators in our cohort, whereas procalcitonin elevation was observed in only 10.3% of patients. These diagnostic yield rates directly support findings from a recent meta‐analysis by Ansert et al. [10], which demonstrated that ESR acts as an excellent biomarker and CRP as an acceptable parameter for identifying osteomyelitis in neuropathic conditions, particularly diabetic foot osteomyelitis. Furthermore, MRI confirmed chronic osteomyelitis in 96.6% of imaged patients in our series, reinforcing its crucial role in delineating bone necrosis and involucrum formation prior to surgical intervention [3, 11].

Microbiologically, our cohort demonstrated a highly complex and diverse profile, yielding 42 distinct microorganisms across 89.7% of cultured patients. While *Staphylococcus aureus* is universally recognized as the predominant single pathogen in acute and chronic hematogenous pediatric osteomyelitis [12–14], our findings reflect a predominant polymicrobial etiology secondary to chronic, open cutaneous ulcers. This microbiological landscape closely mirrors that of adult diabetic foot osteomyelitis rather than typical pediatric bone infections [15–17]. The high frequency of polymicrobial colonization and resistant organisms presents substantial therapeutic challenges, complicating empirical parenteral coverage and necessitating prolonged, culture‐guided antimicrobial regimens [17–19].

Consequently, this combination of severe diagnostic delay, extensive tissue destruction, and polymicrobial infection translated into a high surgical burden [20]. Surgical intervention was required in 86.2% of our patients, with 37.9% ultimately undergoing lower extremity amputation due to refractory infection. Moreover, among patients who maintained regular follow‐up, 100% experienced disease relapses requiring repeated hospitalizations (up to 14 admissions per patient). Rather than reflecting an inherent failure of medical therapy, these high rates of surgical debridement, structural tissue loss, and recurrence illustrate that osteomyelitis in neuropathic children is advanced at the moment of initial presentation [21, 22].

While this study is subject to structural limitations inherent to its retrospective design and single‐center nature (detailed in the Limitations section), these findings provide descriptive observations regarding a challenging patient group. In our experience, pediatric chronic osteomyelitis secondary to underlying neuropathy is characterized by delayed presentation, complex polymicrobial isolates, and substantial surgical burden [6, 23]. Early clinical vigilance by caregivers to identify superficial skin breakdown before bone involvement occurs remains essential to reduce the substantial morbidity associated with this condition.

## 5. Conclusion

In conclusion, the unique microbiological landscape observed in our study underscores the necessity for a vigilant approach to empirical antibiotic selection, the critical role of culture‐guided therapy, and the continuous development of strategic approaches to combat resistant pathogens. These findings provide a strong foundation for re‐evaluating current treatment algorithms and improving clinical outcomes in this vulnerable pediatric population. Early detection of patients with neuropathy, emphasis on protection from trauma and hygiene education, and protection from pressure ulcers are essential steps to be taken to prevent osteomyelitis. We think that the development of osteomyelitis can be prevented by avoiding ulcerations that may occur in these patients by applying and developing surgical techniques. Considering situations such as multiple microorganisms, costly antibiotics, prolonged hospitalizations, and deterioration in patients’ quality of life, it is evident that precautions must be taken before osteomyelitis develops in this disease group.

### 5.1. Limitations of the Study

This study, while addressing an important and challenging clinical problem, has several limitations that should be acknowledged. The sample size of 29 pediatric patients is relatively small, which may limit the statistical power of our findings and their generalizability to the broader pediatric population with neuropathy‐related osteomyelitis. Future research would benefit from multicenter studies with larger cohorts to enhance the robustness of results. The retrospective design of our study, which relied on existing hospital database records, inherently introduces potential biases. Data collection was dependent on the completeness and accuracy of historical patient files, which could lead to missing information or inconsistencies, thereby affecting the accuracy and reliability of our findings. A prospective study design would allow for more standardized data collection and better control over confounding variables. The absence of a control group (pediatric patients with osteomyelitis but without neuropathy) makes it challenging to draw definitive comparisons regarding the unique characteristics, disease progression, and treatment outcomes specific to osteomyelitis in neuropathic children. This limits our ability to establish causality or highlight the distinct impact of neuropathy on the osteomyelitis course. Finally, a major limitation of this study is the high rate of loss to follow‐up, with 18 of 29 patients (62.1%) not maintaining long‐term outpatient monitoring. As a tertiary referral university hospital, many patients were referred from distant geographical regions for acute management (complex surgical intervention and intravenous antimicrobial therapy) and subsequently returned to local medical centers for routine care once stabilized. This substantial attrition introduces potential attrition and selection biases. Specifically, the 100% recurrence rate observed among the 11 patients who completed regular outpatient follow‐up may represent an overestimation of overall recurrence risk, as patients experiencing disease relapses or chronic complications are inherently more likely to return to a tertiary referral center for re‐evaluation and re‐admission.

## Author Contributions

Fatma Kılınc (MD) contributed to the study design, data collection and measurements, statistical analysis, and manuscript preparation. Akif Mirioğlu (MD) contributed to data collection, measurements, and manuscript preparation. Derya Alabaz (MD, Professor) contributed to the study design and manuscript preparation. Ummuhan Cay (MD, Associate Professor) and Ozlem Ozgur Gundeşlioğlu (MD, Professor) contributed to the study design.

## Funding

This research received no specific grant or financial support from any funding agency in the public, commercial, or not‐for‐profit sectors.

## Disclosure

The manuscript has been read and approved by all authors; the requirements for authorship as stated earlier in this document have been met, and each author believes that the manuscript represents honest work.

## Ethics Statement

The study protocol was reviewed and approved by the Çukurova University Hospital Ethics Committee (Approval number: 145/14.06.2024). Because this was a retrospective analysis of records from patient care already provided, the requirement for individual parental informed consent was waived by the committee, in accordance with national regulations governing retrospective chart‐review studies.

## Consent

Please see the Ethics Statement.

## Conflicts of Interest

The authors declare no conflicts of interest.

## Data Availability

The data that support the findings of this study are available on request from the corresponding author. The data are not publicly available due to privacy or ethical restrictions.
